# Derivation of induced pluripotent stem cells in Japanese macaque (*Macaca fuscata*)

**DOI:** 10.1038/s41598-018-30734-w

**Published:** 2018-08-15

**Authors:** Risako Nakai, Mari Ohnuki, Kota Kuroki, Haruka Ito, Hirohisa Hirai, Ryunosuke Kitajima, Toko Fujimoto, Masato Nakagawa, Wolfgang Enard, Masanori Imamura

**Affiliations:** 1 Molecular Biology Section, Department of Cellular and Molecular Biology, Primate Research Institute, Kyoto University, Inuyama, Aichi 484-8506 Japan; 2 Center for iPS Cell Research and Application (CiRA), Kyoto University, Kyoto, 606-8507 Japan; 3 Anthropology and Human Genomics, Department Biology II, Ludwig Maximilians University Munich, Grosshaderner Str. 2, 82152 Planegg-Martinsried, Germany; 4 Department of Life Science, Gakushuin University, Tokyo, 171-8588 Japan

## Abstract

Non-human primates are our closest relatives and are of special interest for ecological, evolutionary and biomedical research. The Japanese macaque (*Macaca fuscata*) has contributed to the progress of primatology and neurosciences over 60 years. Despite this importance, the molecular and cellular basis of the Japanese macaque remains unexplored since useful cellular tools are lacking. Here we generated induced pluripotent stem cells (iPSCs) from skin fibroblasts of the Japanese macaque with Sendai virus or plasmid vectors. The Japanese macaque iPSCs (jm-iPSCs) were established under feeder-free culture conditions, but feeder cells turned out to be essential for their maintenance. The jm-iPSCs formed human iPSC-like flat colonies which were positive for pluripotent antigens including alkaline phosphatase, SSEA4, and TRA-1-81. They also expressed endogenous *OCT3/4*, *SOX2*, *L-MYC*, and *KLF4* and other pluripotent marker genes. The potential to differentiate into all three germ layers and neural stem cells was confirmed by embryoid body and neurosphere formation, respectively. The jm-iPSCs will provide a robust *in vitro* tool for investigating the underlying mechanisms of development and physiology studies with the Japanese macaque.

## Introduction

The Japanese macaque (*Macaca fuscata*) is one species of the Asian macaque monkeys along with rhesus and cynomolgus monkeys. The Japanese macaque - also known as the snow monkey - is native to Japan and is the most northern living non-human primate species. Historically, field researchers have studied social behavior and ecology of the Japanese macaque for more than half a century for religious reasons, leading to the pioneering of primatology in Japan^1–3^. The Japanese macaque also has attracted researcher’s attention in laboratories as a valuable experimental animal model due to their anatomical and physiological similarities to humans, their high cognitive ability, and their gentle and patient character^4^. Especially, the Japanese macaque has contributed to the advance of neuroscience^5^ through the investigations of brain activity and cognition^6–9^, neural pathway and gene expression^10,11^, and spontaneous mutant monkeys^12–15^. From the above, the Japanese macaque has been employed as an excellent primate model with the accumulated knowledge and a potential to integrate the laboratory studies with the field research. However, difficulties in developmental physiology and genetic manipulations *in vivo* restrict in-depth analyses of molecular and cellular mechanisms in the Japanese macaque biology.

Induced pluripotent stem cells (iPSCs) can provide a new strategy to compensate this experimental concern of the Japanese macaque. iPSCs are a new type of pluripotent stem cells that have revolutionized stem cell biology and medicine^16,17^ and have a huge potential for evolutionary and comparative genomic approaches^18–26^. As pluripotent stem cells, iPSCs have a potential to differentiate into all three germ layers (ectoderm, mesoderm, and endoderm) and germ cells both *in vivo* and *in vitro*. Technically, iPSCs are regarded to have four experimental advantages in research. First, iPSCs can be generated from many different types of small biopsies of mammalian species with relatively simple protocols^26–28^. Second, iPSCs have robust and infinite proliferation activity while self-renewing. Third, iPSCs can reconstruct developmental events of interest *in vitro*, providing alternatives for invasive study using animal individuals. Fourth, iPSCs are amenable to genetic manipulations including genome editing. Thus, iPSCs can serve as a potent *in vitro* tool to address the molecular and cellular basis of development and physiology in the Japanese macaque.

In this study, we generated the Japanese macaque iPSCs (jm-iPSCs) from skin fibroblasts by transducing a set of human reprogramming factors with Sendai virus (SeV)^27^ or plasmid^28,29^ vectors. The jm-iPSCs were similar with human iPSCs in colony morphology, marker gene expression, and differentiation potency albeit their growth totally depended on feeder cells. Embryoid body and neurosphere formation cultures induced differentiation of the jm-iPSCs spontaneously into three germ layers or specifically into neural stem cells respectively. The jm-iPSCs will be a powerful *in vitro* counterpart to facilitate the Japanese macaque biology.

## Materials and Methods

### Ethics

All experiments using primate samples in this study were approved by the Animal Care and Use Committee of Kyoto University Primate Research Institute (KUPRI) and were performed in accordance with the Guidelines for Care and Use of Nonhuman Primates (Version 3, 2010) published by KUPRI.

### Generation of jm-iPSCs by Sendai virus vector infection and plasmid vector transduction

Japanese macaque skin fibroblasts (jm1481, 21-year-old female; jm2623, 6-day-old female) were cultured in the 15%FBS/DMEM medium consisting of Dulbecco’s modified Eagle’s medium (DMEM) supplemented with 15% fetal bovine serum (FBS), 0.1 mM non-essential amino acids, 2 mM L-glutamine, 1 mM sodium pyruvate, 0.11 mM 2-mercaptoethanol, and 100 U/ml penicillin and 100 µg/ml streptomycin at 37 °C with 5% CO_2_.

SeV infection was performed using CytoTune-iPS 2.0 L (MBL, DV-0305) with a modified protocol. Briefly, 1 × 10^5^ jm-fibroblasts were incubated in 100 μl of SeV mixture containing KOS(PM)-SeV/TS12ΔF, KLF4-SeV/TSΔF, and L-MYC(HNL)-SeV/TS15ΔF at a multiplicity of infection 5, 5, and 10 each at 37 °C for 1 hour. To assess the successful transduction, CytoTune-EmGFP SeV was also infected. The infected 5 × 10^4^ cells were plated to the iMatrix 511-coated 6-well plates (day 0). The SeV-containing medium was exchanged to fresh 15%FBS/DMEM on day 1. For plasmid transfection, 1 × 10^5^ jm-fibroblasts were transfected with pCXLE-hOct3/4-shp53-F, pCXLE-hSK, pCXLE-hUL^28^ and pCXWB-EBNA1^29^ by Lipofectamine 3000 (Thermo Fisher Scientific, L3000). pCXLE-EGFP was used as a transfection control. After 6 days of culture with 15%FBS/DMEM, 5 × 10^4^ cells were replated to the iMatrix 511 (Matrixome, 389-07364)-coated 35-mm plates.

In both cultures, the medium was exchanged to mTeSR1 (StemCell Technologies, ST0580) on day 7 and medium change was performed every other day. The resulting iPSC-like colonies were mechanically isolated and incubated with TrypLE Select (Life Technologies, 12563029). The cells were plated to iMatrix 511-coated plates and cultured with StemFit (Ajinomoto, AK02N) supplemented with 10 μM of Y-27632 (Wako, 253-00513). After several passages, the jm-iPSCs were maintained on mitomycin-treated SNL feeder cells with StemFit at 37 °C with 5% CO_2_

### Chromosome analysis

The jm-iPSCs at a 70~80% confluency were treated with 50 ng/ml colcemid for 40 minutes. The cells were harvested by TrypLE Express, treated with 0.56% KCl for 20 minutes at room temperature, and fixed with ethanol-acetic acid (3:1) for 10 minutes on ice^30^. Chromosome spreads were prepared with one drop of fixed cell suspension. After treatment with 0.05% IGEPAL CA-630, the chromosome samples were stained with ProLong Diamond Antifade Mountant with DAPI (Thermo Fisher Scientific, P36966). Karyotyping of chromosomes was performed according to the previous report^31^.

### Alkaline phosphatase, rBC2LCN lectin, and immunofluorescence analyses

Alkaline phosphatase staining and rBC2LCN live cell staining was performed using Leukocyte Alkaline Phosphatase kit (Sigma, 86R-1KT) and rBC2LCN-FITC (Wako, 180-02991) respectively, according to the manufacture’s instructions. For immunofluorescence microscopy, cells were fixed with 4% paraformaldehyde, permeabilized with 0.5% Triton-X, blocked with 5% skim milk, and incubated with primary antibodies. The following primary antibodies were used: mouse anti-OCT4 (BD Biosciences, 611202), rabbit anti-NANOG (REPROCELL, RCAB0003P), goat anti-LIN28A (R&D systems, AF3757), rabbit anti-DPPA4 (Abcam, ab154642), mouse anti-SOX2 (R&D systems, MAB2018), rabbit anti-SALL4 (Abcam, ab29112), mouse anti-SSEA4 (Millipore, MAB4304), mouse anti-TRA-1-81 (Millipore, MAB4381), mouse anti-E-CADHERIN (BD Biosciences, 610182), mouse anti-Keratan sulfate (R-10G) (Wako, 011-25811), mouse anti-TUBULIN β3 (BioLegend, MMS-435P), mouse anti-α-SMA (Abcam, ab7817), rabbit anti-VIMENTIN (Abcam, ab92547), goat anti-SOX17 (R&D systems, AF1924), mouse anti-AFP (R&D systems, MAB1369), mouse anti-NESTIN (Millipore, MAB5326), rabbit anti-PAX6 (Wako, 019-27291), rabbit anti-MAP2 (Millipore, AB5622), mouse anti-DREBRIN (Wako, 015-27271) antibodies. Secondary antibodies included Alexa Fluor Plus 488 goat anti-mouse IgG (Thermo Fisher Scientific, A32723), Alexa Fluor 488 donkey anti-goat IgG (Thermo Fisher Scientific, A11055), Alexa Fluor 555 donkey anti-mouse IgG (Abcam, ab150110), Alexa Fluor 555 donkey anti-rabbit IgG (Thermo Fisher Scientific, A31572). Nuclei were stained with 1 µg/ml DAPI.

### RT-PCR and PCR analyses

Total RNA was extracted using RNeasy Plus Mini Kit (Qiagen, 74104) and reverse transcribed using the PrimeScript RT reagent Kit with gDNA Eraser (TaKaRa, RR047A). Genomic DNA was isolated using DNeasy Blood & Tissue Kit (Qiagen, 69506). RT-PCR and PCR analyses were performed with Ex Taq Hot Start Version (TaKaRa, RR006A). All experiments were performed semiquantitatively at three different escalation cycles, and only representative images are shown in the result.

### *In vitro* differentiation culture

For embryoid body (EB) formation, jm-iPSCs were dissociated and transferred to 6-well low attachment culture plates (corning) at 1 × 10^6^ cells/ml in 15%FBS/DMEM. After 2 weeks culture, the EBs were transferred to Geltrex-coated plates and cultured at an adhesion condition with 15%FBS/DMEM for another 3 weeks.

For directed differentiation into neural stem cells, jm-iPSCs were transferred to 6-well low attachment plates in KBM Neural Stem Cell medium (Kohjin Bio, 16050200) supplemented with 2 μM dorsomorphin (Cayman, 11967), 10 μM SB431542 (Cayman, 13031), and 1 × B-27 (Gibco, 17504-044) at 1.5 × 10^5^ cells/ml. Soon after iPSC dissociation, 10 μM Y-27632 was added to the medium. On day 1 and 4, 3 ng/ml Stembeads FGF2 (StemCultures, SB500) were added into the medium and the medium was half-exchanged on day 4. To induce neuronal differentiation, 1-week old neurospheres were plated onto Geltrex-coated culture plates and cultured in KBM Neural Stem Cell medium without FGF2 and EGF but supplemented with 1 × B-27 for 2 weeks.

## Results

**Figure 1:**
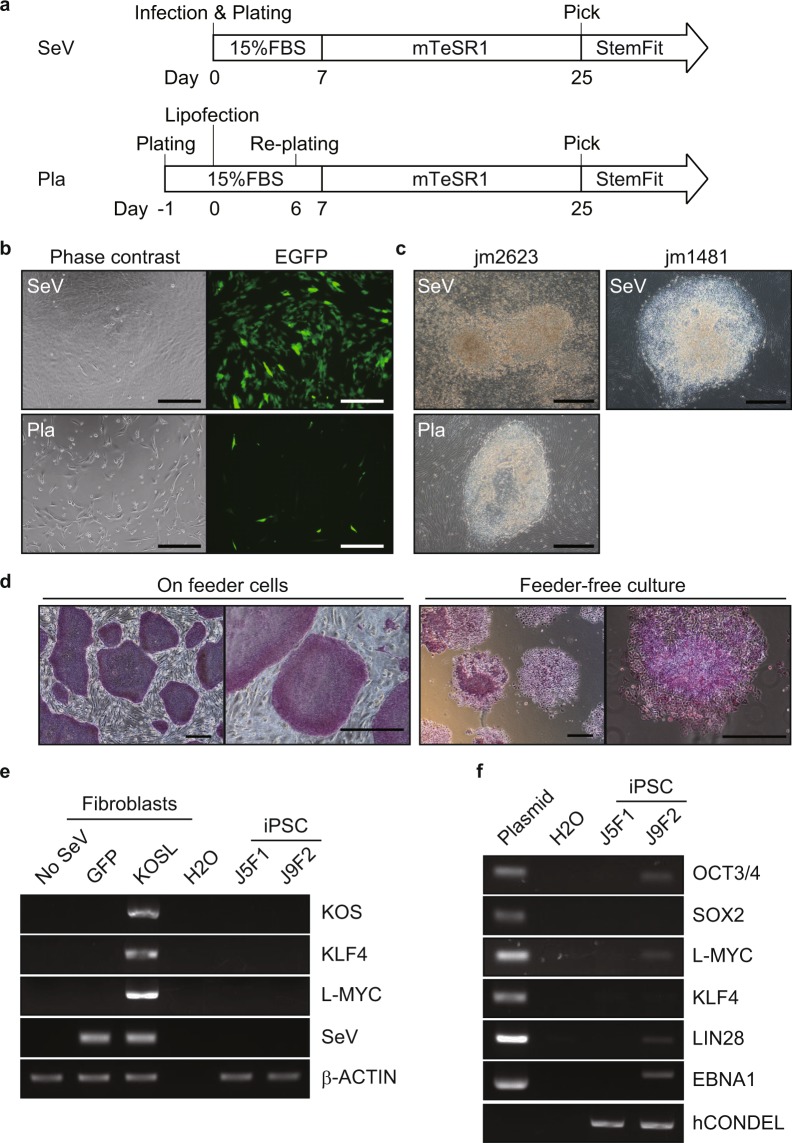
Generation of jm-iPSCs from skin fibroblasts (**a**). Schematic design of jm-iPSC generation. SeV, Sendai virus system. Pla, plasmid vector system. (**b**) The expression of GFP transgenes in jm-fibroblasts (jm2623) on day 3 after SeV (upper) and plasmid vector (lower) transduction. Scale bar; 100 µm. (**c**) Phase contrast images of iPSC-like colony morphologies emerged from jm2623 (day 25, upper; SeV, lower; plasmid) and jm1481 fibroblasts (day 32, SeV). Scale bar; 100 µm. (**d**) Phase contrast images of jm-iPSCs (J5F1, derived from SeV-jm1481) grown on feeder cells or under feeder-free culture after staining for alkaline phosphatase. Scale bar; 500 µm. (**e**) RT-PCR analysis of SeV vectors. Jm-fibroblasts (jm1481) infected with SeV vectors carrying GFP or reprogramming factors (KOSL, day 3) were used as positive controls of SeV vectors. β-ACTIN was examined as an internal control. Full-length gels are presented in Supplementary Figure S1. (**f**) Genomic PCR analysis of plasmid transgenes. Each plasmid vector carrying reprogramming factors and water were used as a positive and negative control, respectively. An hCONDEL region was examined as an internal control. Full-length gels are presented in Supplementary Figure S1.

To generate jm-iPSCs from primary skin fibroblasts (jm1481, jm2623), we applied two methods to transduce the reprogramming factors with SeV^27^ and plasmid^28,29^ vectors (Fig. 1a). Human POU5F1 (also known as OCT3/4), SOX2, KLF4, and L-MYC were transduced into jm-fibroblasts by SeV infection, while plasmid vectors bearing human OCT3/4, SOX2, KLF4, LIN28, L-MYC, and shRNA for *TP53* were transfected with an additional EBNA1 plasmid by lipofection. In both cultures, the expression of GFP transgenes showed successful gene transduction into jm-fibroblasts albeit the transduction efficiency was much lower in the plasmid transfection (Fig. 1b). iPSC-like colonies appeared from jm2623 fibroblasts within 15 days after SeV infection and 25 days after plasmid transfection. Since jm1481 fibroblasts had a tendency to detach within 3 weeks after vector transduction, we re-plated the cells to a new plate on day 19. Human iPSC-like flat and clear-edged colonies were mechanically dissected and plated onto new dish on day 25 (jm2623 fibroblasts) or day 32 (jm1481 fibroblasts) (Fig. 1c).

Although the jm-iPSC colonies emerged under feeder-free culture, feeder cells were required to maintain the jm-iPSCs in an undifferentiated state. The jm-iPSCs grown on feeder cells formed flat and tightly packed colonies with sharp edges, while cells under feeder-free culture gradually exhibited loose colonies showing a heterogenous alkaline phosphatase staining after serial passages (Fig. 1d). Mouse SNL cells or autologous jm-fibroblasts were effective as feeder cells therefore we utilized SNL cells because of its technical convenience. The jm-iPSCs could be expanded for more than 50 passages.

For characterization of the jm-iPSCs established, we used two different jm-iPSC lines J5F1 (derived from SeV-jm1481) and J9F2 (derived from plasmid-jm2623). SeV vectors replicate their RNA genomes in the cytoplasm of infected cells. During reprogramming, SeV vectors are gradually diluted but can sometimes be detected even in established iPSCs^27^. In J5F1 jm-iPSCs, no residual SeV vectors were detected by RT-PCR (Fig. 1e). We also performed PCR to detect plasmid transgenes in the established iPSC lines and identified a genomic integration of OCT3/4, L-MYC, LIN28, and EBNA1 in J9F2 jm-iPSCs (Fig. 1f).

**Figure 2:**
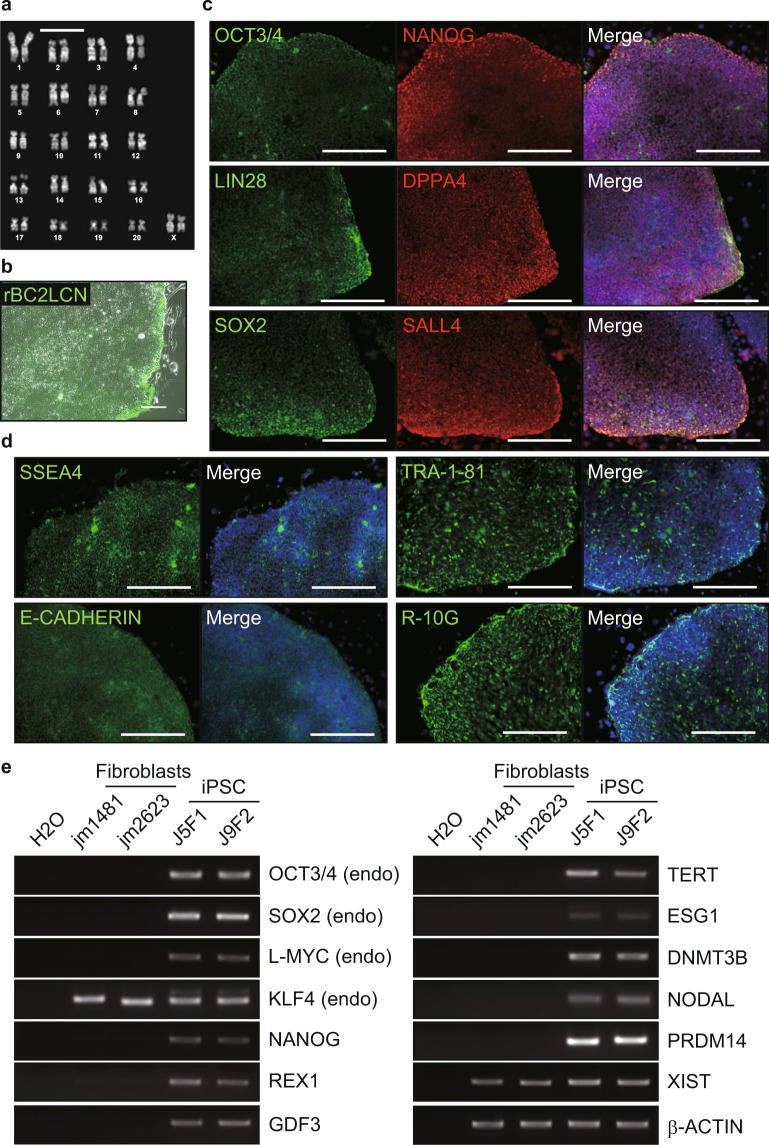
Molecular characterization of jm-iPSCs (J5F1) (**a**). Chromosomal analysis of jm-iPSCs (J5F1). Scale bar; 10 µm. (**b**) Live staining of jm-iPSCs (J5F1) with rBC2LCN lectin. Scale bar; 200 µm. (**c**) Immunofluorescence analyses of pluripotency-associated proteins OCT4, NANOG, LIN28, DPPA4, SOX2, and SALL4. (**d**) Immunofluorescence analyses of pluripotency-associated cell surface antigens SSEA4, TRA-1-81, E-CADHERIN, and R-10G. Nuclei were counterstained with DAPI. Scale bar; 250 µm. (**e**) RT-PCR analyses of pluripotency-associated genes. β-ACTIN was examined as an internal control, and water was used as a negative control. Full-length gels are presented in Supplementary Figure S1.

The jm-iPSCs had normal karyotype (42 XX) (Fig. 2a, Supplementary Figure S3a). The jm-iPSCs were strongly positive for rBC2LCN lectin^32^ staining (Fig. 2b, Supplementary Figure S3b). Immunofluorescence analyses showed that the jm-iPSCs expressed other pluripotency intracellular antigens (OCT3/4, NANOG, LIN28, DPPA4, SOX2, SALL4) (Fig. 2c, Supplementary Figure S3c) and cell surface antigens (SSEA4, TRA-1-81, E-CADHERIN) (Fig. 2d, Supplementary Figure S3d). R-10G sugar chain epitope^33^ was also detected in the jm-iPSCs, indicating that the jm-iPSCs were highly reprogrammed pluripotent stem cells. RT-PCR revealed the expression of a set of pluripotency-associated genes, such as *REX1*, *GDF3*, and *TERT*, in the jm-iPSCs but not in the parental fibroblasts (Fig. 2e). The endogenous expression of *OCT3/4*, *SOX2*, *L-MYC*, and *KLF4* was also induced in the jm-iPSCs. The expression of *XIST* showed that X chromosome reactivation did not occur in these female jm-iPSCs, suggesting their primed pluripotency.

**Figure 3:**
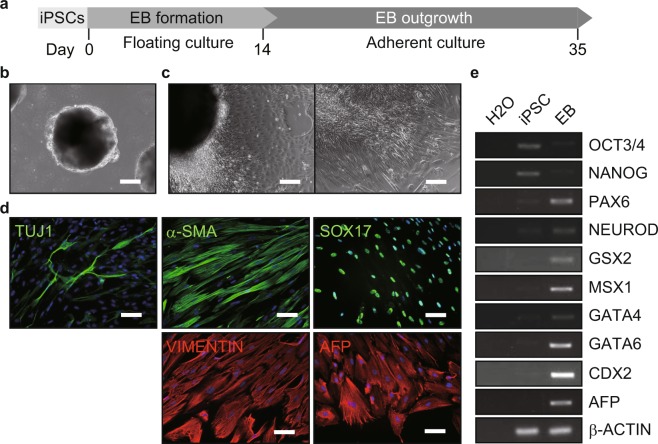
Differentiation potency of jm-iPSCs (J5F1) into three germ layers (**a**). Schematic design of embryoid body (EB)-mediated differentiation of jm-iPSCs. (**b**) jm-iPSC (J5F1)-derived EBs after 2-week floating culture. Scale bar; 200 µm. (**c**) Outgrowth of jm-iPSC (J5F1)-derived EBs at 3-week adherent culture. Representative images of differentiated cells are shown. Scale bar; 200 µm. (**d**) Immunofluorescence analyses of ectoderm (TUJ1), mesoderm (α-SMA, VIMENTIN), endoderm markers (SOX17, AFP) in the EB outgrowth. Nuclei were counterstained with DAPI. Scale bar; 100 µm. (**e**) RT-PCR analysis of differentiation marker genes in the floating EBs. β-ACTIN was examined as an internal control, and water was used as a negative control. Full-length gels are presented in Supplementary Figure S2.

**Figure 4:**
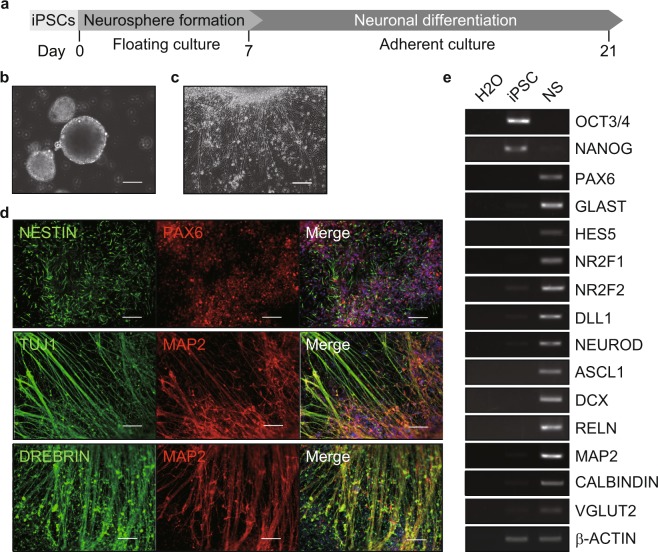
Directed differentiation of jm-iPSCs (J5F1) into neural cells (**a**). Schematic design of neural differentiation of jm-iPSCs. (**b**) Neurospheres of jm-iPSCs (J5F1) after 1-week floating culture. Scale bar; 100 µm. (**c**) Neuronal differentiation of neurospheres after 2-week adherent culture. Scale bar; 200 µm. (**d**) Immunofluorescence analyses of neural stem cell (NESTIN, PAX6) and neuron markers (TUJ1, MAP2, DREBRIN) in 2-week neuronal differentiation culture of neurospheres. Nuclei were counterstained with DAPI. Scale bar; 100 µm. (**e**) RT-PCR analysis of neural marker genes. β-ACTIN was examined as an internal control, and water was used as a negative control. NS, neurospheres after 2-week neuronal differentiation culture. Full-length gels are presented in Supplementary Figure S2.

Differentiation potency of jm-iPSCs was examined by embryoid body (EB) and neurosphere formation cultures. In EB formation culture, jm-iPSCs formed ball-like structures at a floating condition (Fig. 3a,b, Supplementary Figure S4a). When transferred to the adherent culture, the EBs expanded and various kinds of differentiated cells appeared spontaneously (Fig. 3c, Supplementary Figure S4b). Immunofluorescence analysis revealed differentiation to the ectoderm (TUJ1), mesoderm (α-SMA, VIMENTIN), and endoderm (SOX17, AFP) lineage in the EB outgrowth (Fig. 3d, Supplementary Figure S4c). The expression of the three germ layer markers in the EBs was also detected by RT-PCR analysis (Fig. 3e, Supplementary Figure S4d). However, compared to J5F1 jm-iPSCs, J9F2 jm-iPSCs were likely to have less differentiation potency. Then, neurosphere formation culture^34–36^ demonstrated the ability to directed differentiation into neural stem cells (Fig. 4a). The jm-iPSCs formed neurospheres in the presence of chemical dual SMAD inhibitors^37^ during 1-week floating culture (Fig. 4b, Supplementary Figure S5a). The neurospheres were then cultured at a neuronal differentiation condition for another 2 weeks (Fig. 4c, Supplementary Figure S5b). Immunofluorescence analysis detected the efficient production of neural stem cells (NESTIN^+^, PAX6^+^) and neurons (TUJ1^+^, MAP2^+^, DREBRIN^+^) in the differentiated neurospheres (Fig. 4d, Supplementary Figure S5c). Again, RT-PCR analysis also revealed the expression of neural stem/progenitor cells (GLAST, ASCL1 etc.) and neuron markers (DCX, VGLUT2 etc.) after neuronal differentiation of the neurospheres (Fig. 4e, Supplementary Figure S5d).

## Discussion

Here, we generated jm-iPSCs from adult and neonate skin fibroblasts by transducing human reprogramming factors with Sendai virus and plasmid vectors. Once established, the jm-iPSCs formed tight and flat colonies similarly to human iPSCs and grew over 50 passages. They expressed the pluripotency-associated genes and exhibited differentiation potency into all three germ layers. Basic molecular and cellular properties of the jm-iPSCs seemed to be similar with those of human iPSCs. To date, numerous number of methods have been developed to induce directed differentiation from human iPSCs into cell types of interest *in vitro*, and, among them, neurosphere formation culture^36,37^ could be applied to the jm-iPSCs to derive neural stem cells (Fig. 4, Supplementary Figure S5). Thus, *in vitro* differentiation protocols for human iPSCs are expected to be applicable to the jm-iPSCs. Experimental platforms have been set up to study *in vitro* developmental biology of the Japanese macaque and comparative Evo-Devo studies among primate species including human.

Although jm-iPSCs could be cultured basically referring to human iPSC protocols, Japanese macaque fibroblasts were not highly competent for lipofection-based gene transduction (Fig. 1b). In addition, feeder cells were necessary for long term maintenance of the jm iPSCs. For feeder-free culture of the jm-iPSCs, we used laminin-511 E8 coating in combination with StemFit medium that enables efficient feeder-free expansion of human iPSCs even after single cell dissociation^38^. We further assessed additional molecules (ID-8^39^, GF109203X^40^, increased concentration of FGF2 etc.) or other coating reagents (Geltrex etc.), but none of them improved the feeder-free culture of jm-iPSCs (data not shown). Therefore, current feeder-free cultures that have devised for human iPSCs are not sufficient to maintain jm-iPSCs. It remains unclear whether this is due to species-specific cell culture requirements or cellular properties including reprogramming quality. To achieve a conventional feeder-free culture of jm-iPSCs, further investigations of culture conditions, including optimization for macaque monkeys rather than human, will be needed.

Mouse and human iPSC studies have revealed that reprogramming efficiency declines in a donor age-dependent manner^41^. Indeed, albeit jm-iPSCs could be generated from 6-day-old neonate (jm2623) and 21-year-old adult (jm1481) donor monkeys, the neonate showed a much higher amount of iPSC colonies upon reprogramming than the adult macaque (data not shown). It is also suggested that age-related genetic and epigenetic signatures inherited from donor cells may influence the quality of iPSCs^42^. When we compared the jm-iPSC lines J5F1 and J9F2, the differentiation potency seemed to be higher in adult-derived J5F1 than neonate-derived J9F2 iPSCs (Fig. 3, Supplementary Figure S4). Considering the low transduction efficiency and the genomic integration of transgenes in the plasmid transfection-derived iPSCs (Fig. 1b,f), the differences are likely to be explained by the transduction method and subsequent reprogramming quality rather than the donors age. To assess the effects of donor age and gene transduction methods on reprogramming in the Japanese macaque, it is necessary to characterize several jm-iPSC lines derived from various donors with different ages and reprogramming methods.

To date, several spontaneous mutant monkeys have been found in the Japanese macaque populations. For example, a child monkey in our institute was characterized by significant premature aging that exhibited common symptoms with human progeria, including deep skin wrinkles, cataract, poikiloderma, and shrinkage of cerebral cortex^12^. To identify the genes associated with these phenotypes, genomic DNA sequencing of this monkey was performed. However, no mutations in known genes that are responsible for the major human progeroid syndromes were detected so that the molecular basis underlying the premature aging remained unclear. In another case, a monkey that exhibited an autism spectrum disorder was reported^14^. This monkey exhibited impaired social ability, repetitive behaviors, and less neurons responsive to other monkey’s action. Genomic analyses of copy number variation and exome sequencing identified many mutations unique to this monkey including two genes associated with neuropsychiatric disorders in humans; however it remains to be determined whether these genes are actually causal for the phenotype. To fill the gap between phenotype and genotype, the jm-iPSCs from mutant monkeys are useful for in-depth, comprehensive analyses as demonstrated by disease-specific iPSCs in human^43^. Directed differentiation of mutant monkey jm-iPSCs enables researchers to model the pathophysiology and investigate its underlying mechanisms *in vitro*. Furthermore, functional assays of genetically manipulated candidate genes will give insight to their contribution to molecular circuits and cellular properties. This approach can be applied to wild type monkeys and a wide range of different primates as well, and intensively support neurosciences from the point of view of molecular, cellular, and developmental biology.

A recent study reported that approximately 60% of non-human primate species is under the risk of extinction and approximately 75% of the populations are decreasing in size^44^. To address this issue, multilateral efforts and solutions including preservation of their habitats or artificial breeding are needed. Given the technical advantages of iPSCs which are easy to generate and proliferate infinitely, the iPSC technology will serve as a backup system to preserve divergent bio-resources. For example, the establishment of a primate iPSC bank as a ‘frozen zoo’ is useful for keeping invaluable genetic materials in a viable condition^45^. Furthermore, considering recent progresses of germ cell production from iPSCs^46^, iPSCs-derived germ cells are likely to be a more direct means for future assisted reproduction. Primate iPSCs would provide a new strategy for conservation and reproduction of primate species, and the Japanese macaque and jm-iPSCs could be a good model for this challenge. This is also helpful to preserve bio-resources of the rare spontaneous mutant monkeys that are found incidentally in populations.

In conclusion, we generated iPSCs from the Japanese macaque fibroblasts with a reprogramming strategy optimized for human iPSCs. This new *in vitro* system would contribute to further development of primatology and neurosciences by facilitating the molecular and cellular understanding of Japanese macaque.

## Electronic supplementary material

Supplementary Figures
