# Supplementary material for: Derivation of induced pluripotent stem cells in Japanese macaque (Macaca fuscata)
Source: Sci Rep. 2018 Aug 15;8:12187. doi: 10.1038/s41598-018-30734-w (PMC6093926; doi:10.1038/s41598-018-30734-w)
Supplement: Supplementary file 1 — Supplementary Figures [file 41598_2018_30734_MOESM1_ESM.pdf]

Derivation of induced pluripotent stem cells in Japanese macaque (*Macaca fuscata*)

Risako Nakai, Mari Ohnuki, Kota Kuroki, Haruka Ito, Hirohisa Hirai, Ryunosuke Kitajima, Toko Fujimoto, Masato Nakagawa, Wolfgang Enard, Masanori Imamura

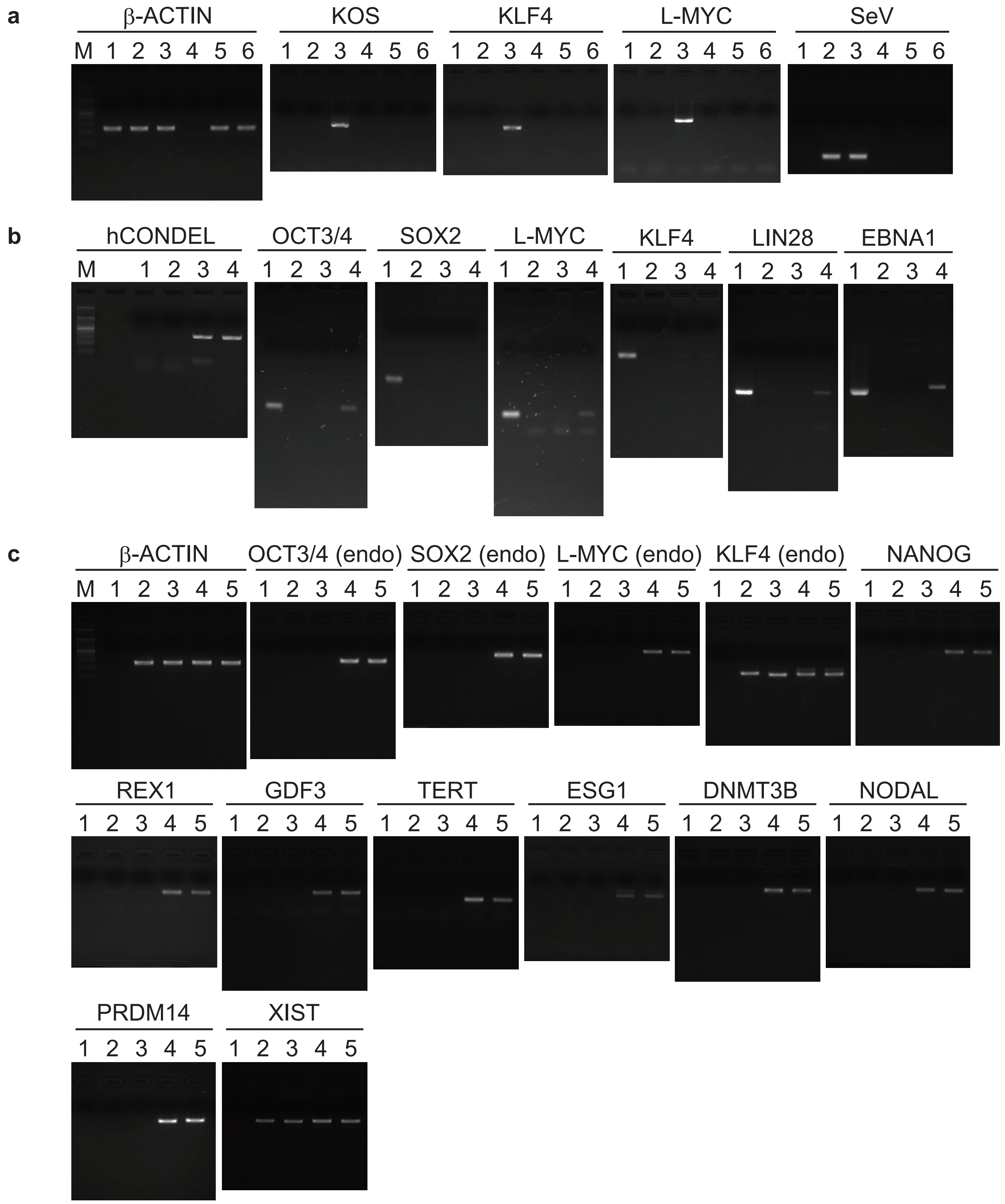

Supplementary Figure S1.

Full-length gels of the data in the main Figure 1 and 2. a. Full-length gels of the data in the main Figure 1e. Lanes 1: jm-fibroblast without SeV; 2: jm-fibroblast with GFP SeV; 3: jm-fibroblast with KOSL SeV; 4: H2O; 5: jm-iPSC (J5F1); 6: jm-iPSC (J9F2). b. Full-length gels of the data in the main Figure 1f. Lanes 1: Plasmid vectors; 2: H2O; 3: jm-iPSC (J5F1); 4: jm-iPSC (J9F2). c. Full -length gels of the data in the main Figure 2. Lanes 1: H2O; 2: jm-fibroblast (jm1481); 3: jm -fibroblast (jm2623); 4: jm-iPSC (J5F1); 5: jm-iPSC (J9F2). M: DNA ladder marker.

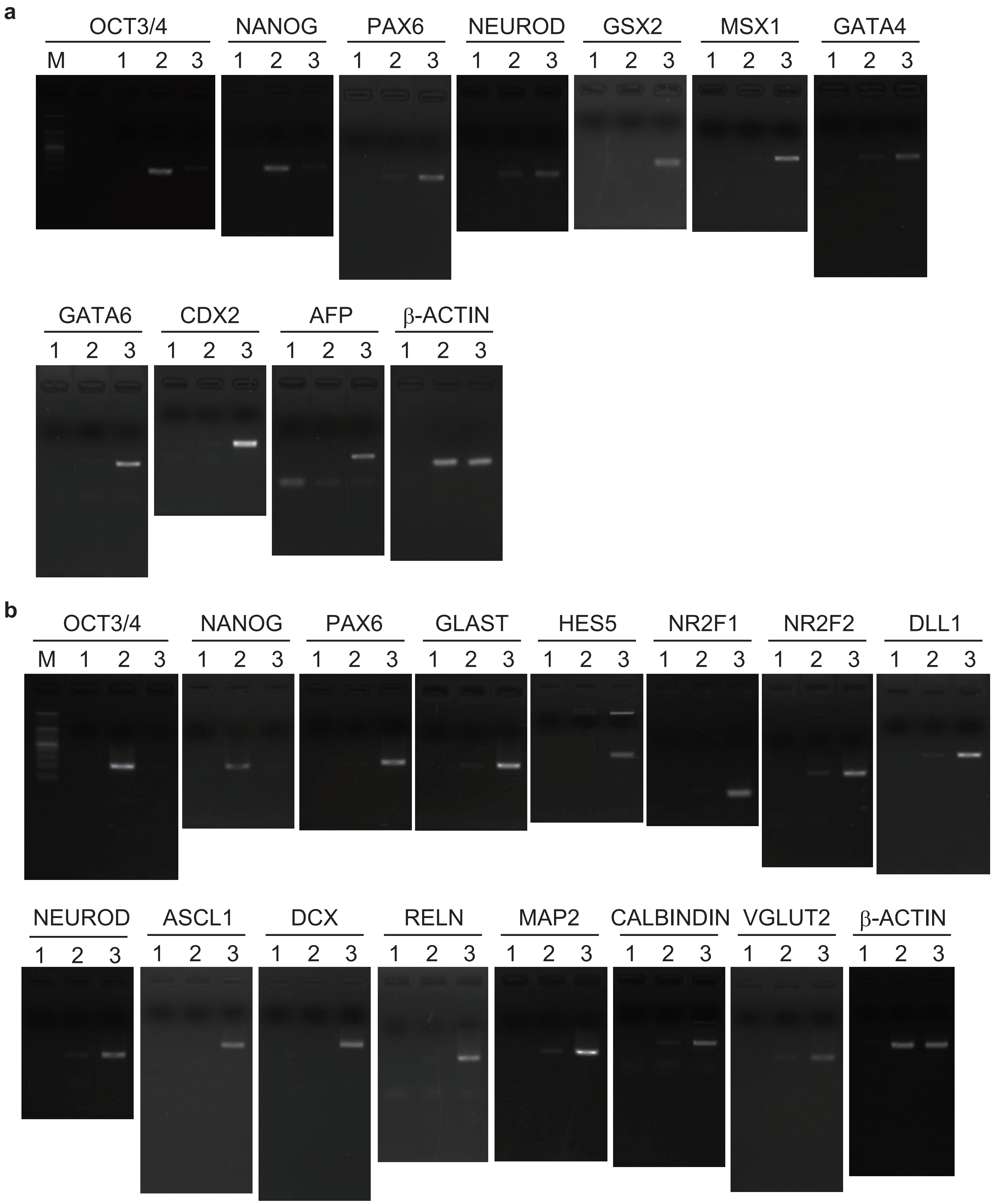

Supplementary Figure S2.

Full-length gels of the data in the main Figure 3 and 4. a. Full-length gels of the data in the main Figure 3. Lanes 1: H2O; 2: undifferentiated jm-iPSC; 3: jm-iPSC-derived EB. b. Full-length gels of the data in the main Figure 4. Lanes 1: H2O; 2: undifferentiated jm-iPSC; 3: jm-iPSC-derived neurosphere. M: DNA ladder marker.

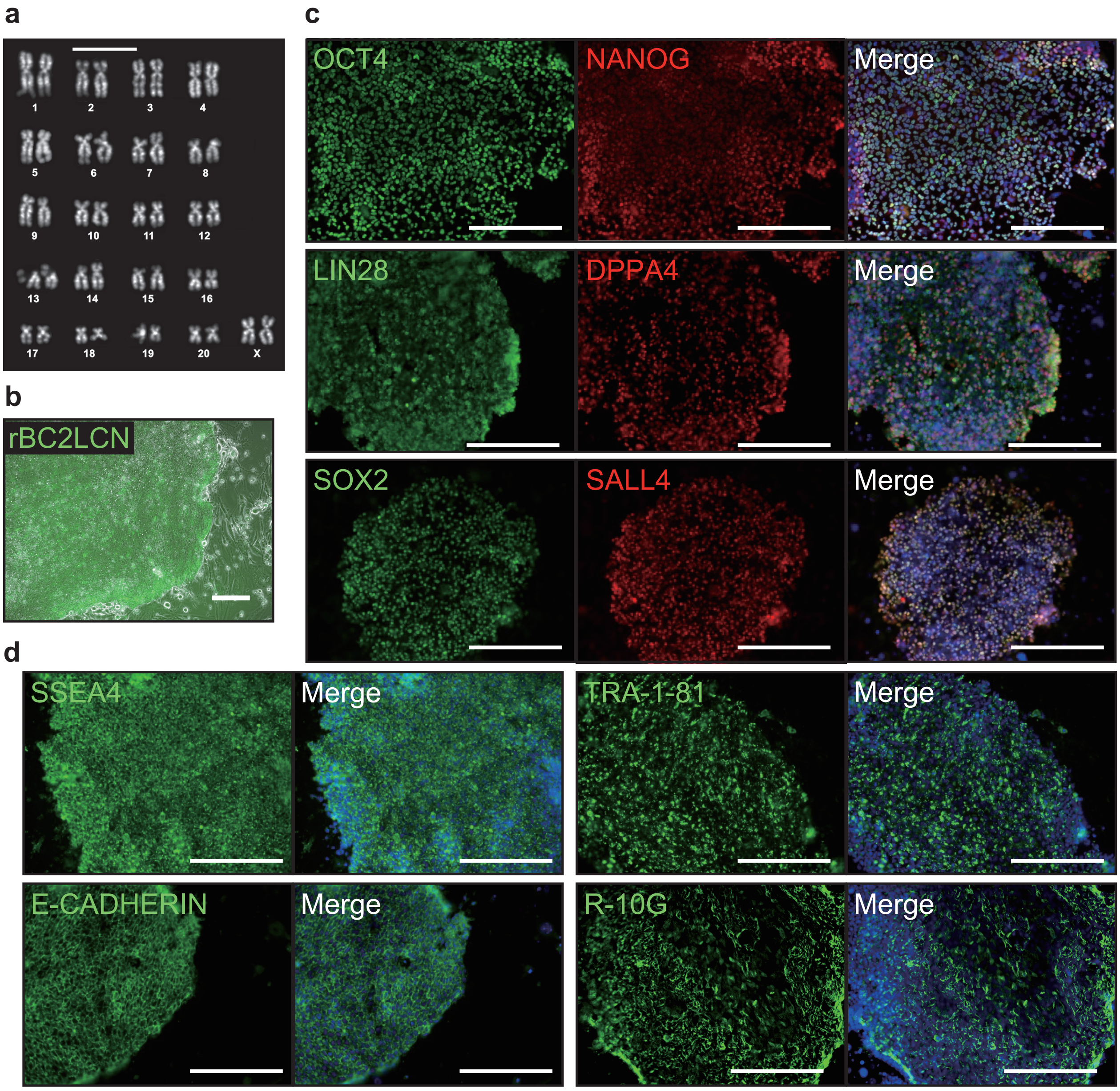

Supplementary Figure S3.

Molecular characterization of jm-iPSCs (J9F2). a. Chromosomal analysis of jm-iPSCs (J9F2). Scale bar; 10 µm. b. Live staining of jm-iPSCs with rBC2LCN lectin. Scale bar; 200 µm. c. Immunofluorescence analyses of pluripotency-associated proteins OCT4, NANOG, LIN28, DPPA4, SOX2, and SALL4. d. Immunofluorescence analyses of pluripotency-associated cell surface antigens SSEA4, TRA-1-81, E-CADHERIN, and R-10G. Nuclei were counterstained with DAPI. Scale bar; 250 µm.

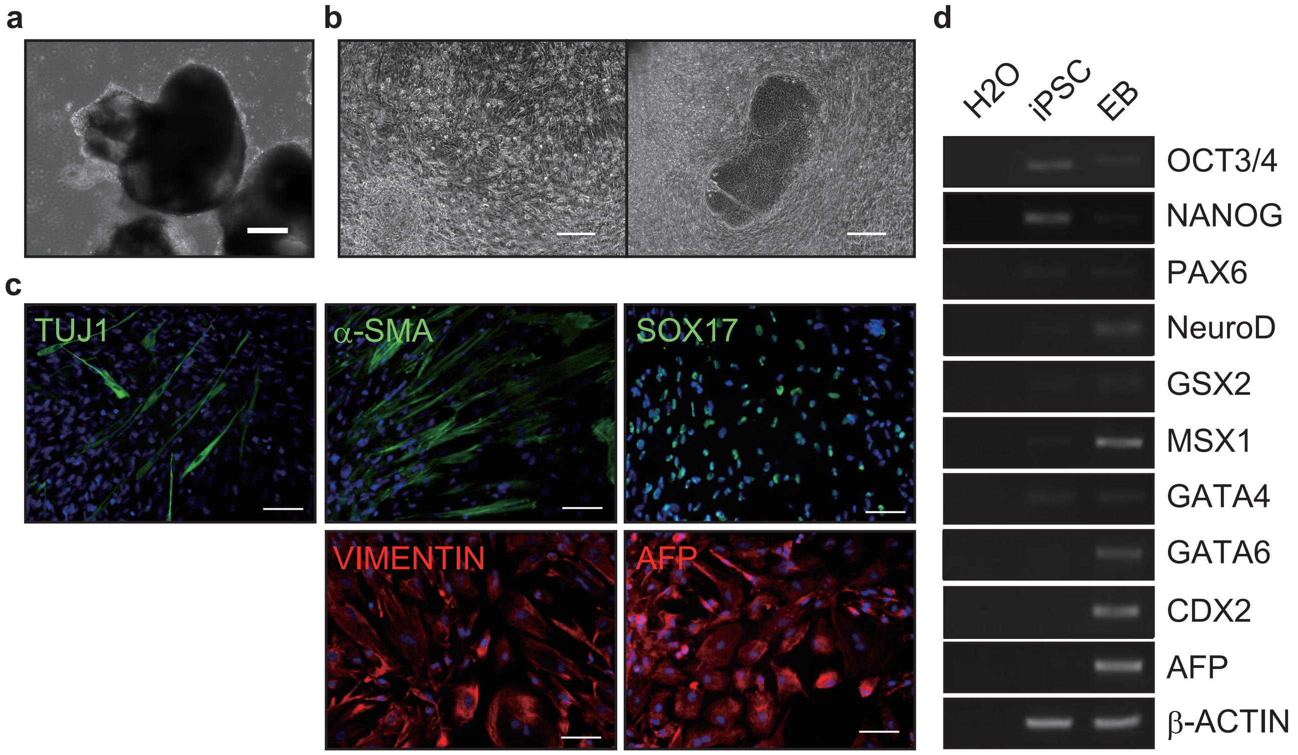

Supplementary Figure S4.

Differentiation potency of jm-iPSCs (J9F2) into three germ layers. a. jm-iPSC (J9F2)-derived EBs after 2-week floating culture. Scale bar; 200 µm. b. Outgrowth of jm-iPSC (J9F2)-derived EBs at 3-week adherent culture. Representative images of differentiated cells are shown. Scale bar; 200 µm. c. Immunofluorescence analyses of ectoderm (TUJ1), mesoderm (α-SMA, VIMENTIN), endoderm markers (SOX17, AFP) in the EB outgrowth. Nuclei were counterstained with DAPI. Scale bar; 100 µm. d. RT-PCR analysis of differentiation marker genes in the floating EBs. β-ACTIN was examined as an internal control, and water was used as a negative control. Full-length gels are presented in Supplementary Figure S6.

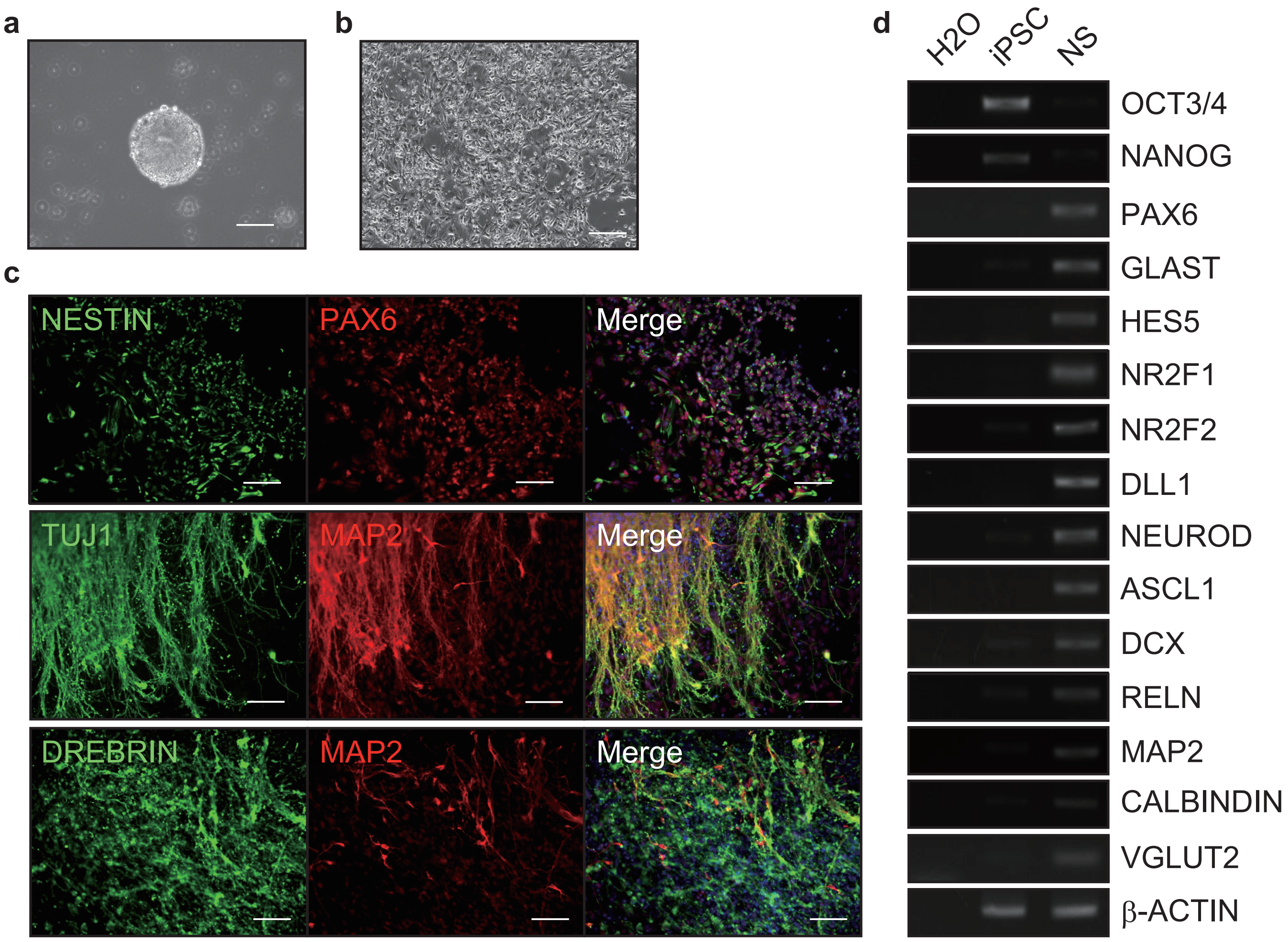

Supplementary Figure S5.

Directed differentiation of jm-iPSCs (J9F2) into neural cells. a. Neurospheres of jm-iPSCs (J9F2) after 1-week floating culture. Scale bar; 100 μm. b. Neuronal differentiation of neurospheres after 2-week adherent culture. Scale bar; 200 μm. c. Immunofluorescence analyses of neural stem cell (NESTIN, PAX6) and neuron markers (TUJ1, MAP2, DREBRIN) in 2-week neuronal differentiation culture of neurospheres. Nuclei were counterstained with DAPI. Scale bar; 100 μm. d. RT-PCR analysis of neural marker genes. β-ACTIN was examined as an internal control, and water was used as a negative control. NS, neurospheres after 2-week neuronal differentiation culture. Full-length gels are presented in Supplementary Figure S6.

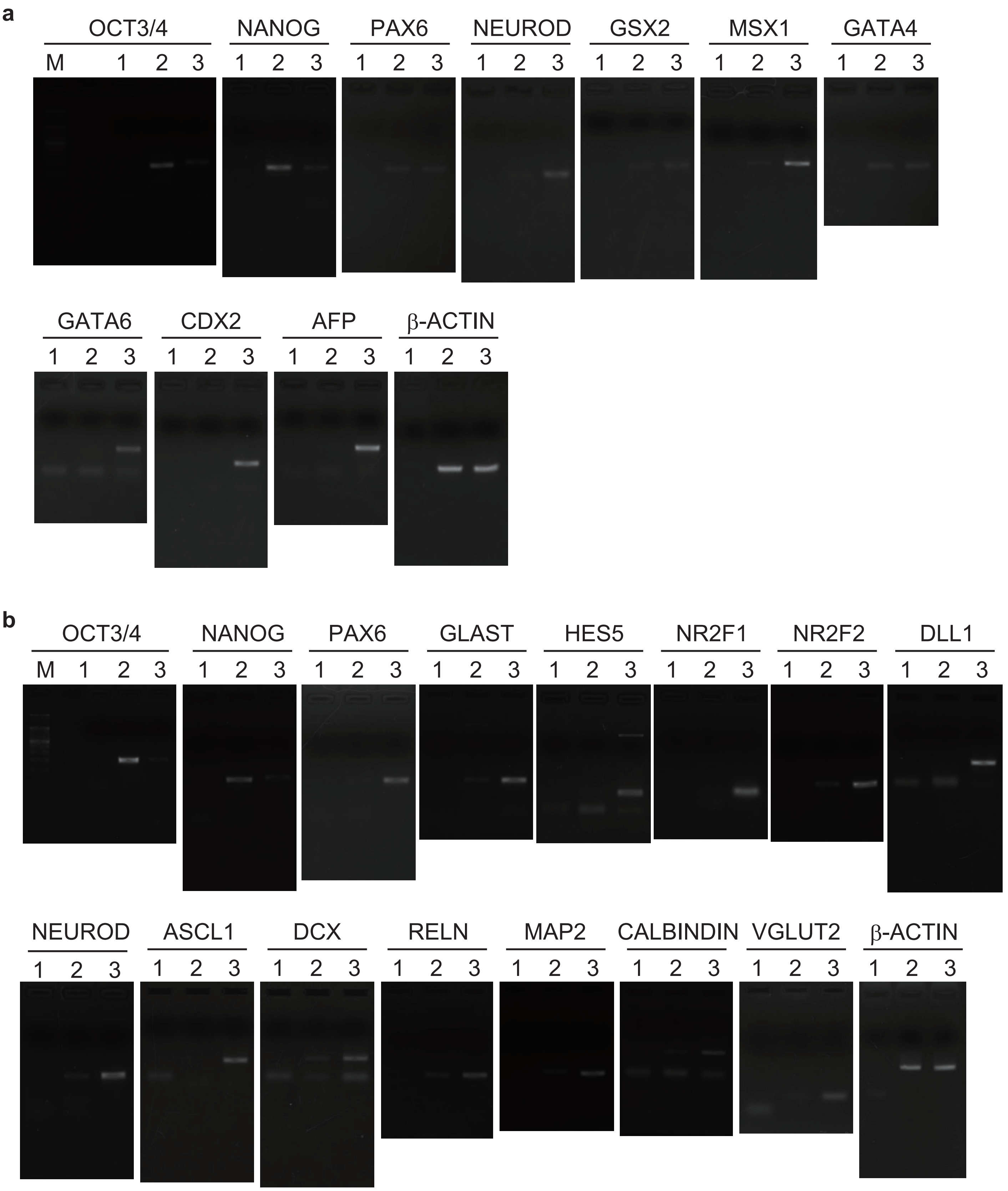

Supplementary Figure S6.

Full-length gels of the data in the Supplementary Figure S4 and S5. a. Full-length gels of the data in the Supplementary Figure S4. Lanes 1: H2O; 2: undifferentiated jm-iPSC; 3: jm-iPSC-derived EB. b. Full-length gels of the data in the Supplementary Figure S5. Lanes 1: H2O; 2: undifferentiated jm-iPSC; 3: jm-iPSC-derived neurosphere. M: DNA ladder marker.
